# Patent foramen ovale closure vs. medical therapy alone after cryptogenic stroke in China: A cost-effectiveness analysis

**DOI:** 10.3389/fpubh.2022.1016854

**Published:** 2022-11-03

**Authors:** Na Wei, Bo Liu, Meijuan Ma, Xuejun Zhang, Wei Zhang, Fangxia Hou, Fuqiang Liu, Xiangyou Yu

**Affiliations:** ^1^Department of Cardiology, Shaanxi Provincial People's Hospital, Xi'an, China; ^2^Department of Endocrinology, Shaanxi Provincial People's Hospital, Xi'an, China

**Keywords:** patent foramen ovale closure, PFO, PFO closure, cryptogenic stroke, patent foramen ovale

## Abstract

**Background:**

In 2019, there were 28. 76 million patients with stroke in China, with ~25% of them suffering from cryptogenic stroke (CS). Patent foramen ovale (PFO) is related to CS, and PFO closure can reduce recurrent stroke. To date, no study has investigated the cost-effectiveness of PFO closure vs. medical therapy among such populations in China.

**Methods:**

A Markov model with a cycle length of 3 months was established to compare the 30-year cost-effectiveness of PFO closure and medical therapy. The transition probability of recurrent stroke was derived from the RESPECT study, and the costs and utility were obtained from domestic data or studies conducted in China. The primary outcome of this study was the incremental cost-effectiveness ratio (ICER), which represents the incremental cost per quality-adjusted life year (QALY). PFO closure was considered cost-effective if the ICER obtained was lower than the willingness-to-pay (WTP) threshold of 37,654 USD/QALY; otherwise, PFO closure was regarded as not being cost-effective. One-way and probabilistic sensitivity analyses were performed to test the robustness of the results.

**Results:**

After a simulation of a 30-year horizon, a cryptogenic stroke patient with PFO was expected to have QALY of 13.15 (15.26 LY) if he received PFO closure and a corresponding value of 11.74 QALY (15.14 LY) after medical therapy. The corresponding costs in both cohorts are US $8,131 and US $4,186, respectively. Thus, an ICER of 2783 USD/QALY and 31264 USD/LY was obtained, which is lower than the WTP threshold. One-way and probabilistic sensitivity analyses showed that the results were robust.

**Conclusion:**

With respect to the WTP threshold of three times per capita GDP in China in 2021, PFO closure is a cost-effective method for Chinese cryptogenic stroke patients with PFO, as shown in the 30-year simulation.

## Introduction

Stroke is a significant public health problem that leads to early death and long-term disability (1). Although age-standard mortality rates have decreased in the past two decades, the absolute number of population and stroke-related deaths is increasing, especially in low- and middle-income countries (2). It was estimated that ~7.2 million adults in America self-report having had a stroke (3) and that 28.76 million people were reported to have the condition in China (4).

It was reported that, among the total number of ischemic strokes, cryptogenic stroke (CS) accounted for about 25% of all stroke cases, which may climb up to 50% in younger patients with stroke (5, 6). CS is an exclusive diagnosis referring to a stroke whose cause cannot be determined after extensive evaluation using various modern examination methods (7). However, extensive research found that patent foramen ovale (PFO) is related to CS, and paradoxical embolization through PFO is a significant cause of undefined cerebral infarction in young- and middle-aged patients (8, 9). Each year, there are ~18,000 adults under 60 years of age in the United States and 345,000 patients worldwide with PFO and embolic stroke of undetermined source (10). Although there is no exact number of CS patients with PFO in China, the number is estimated to be large, given the large population of patients with stroke in China.

A meta-analysis including six randomized controlled trials (RCT) showed that transcatheter PFO closure could reduce the risk of recurrent CS by 58% in patients under 60 years of age (11). Similarly, in a Chinese retrospective cohort study, the 3-year cumulative stroke recurrence rates were 11.48% and 0% in the medical therapy and PFO closure groups of CS patients with PFO, respectively (12). He et al. analyzed data from a hospital in China. They found that PFO closure was safe with no procedure-related deaths, strokes, or transient ischemic attacks (TIA), and the annual risk of recurrent ischemic stroke or TIA was 0.457%, comparable to the rate of 0.58% reported in the RESPECT study (13). Given the potential benefit of PFO closure in patients with CS, the Chinese expert consensus on the preventive closure of PFO recommended PFO closure for patients with CS (6), and it has been reported that there were over 21,000 PFO closure procedures in China in 2021 (14).

Cost-effectiveness analysis is a method that balances the cost and benefit of a new treatment compared to that of the traditional treatment. The incremental cost is defined as the difference between the cost of a new treatment and that of the traditional treatment, and incremental effectiveness is the difference between the effectiveness of a new treatment and that of the traditional treatment. The incremental cost-effectiveness ratio (ICER) is the ratio of incremental costs to incremental effectiveness. The new treatment is considered cost-effective if the ICER is lower than the willingness-to-pay (WTP) threshold, a parameter often set by the government or the Association for Pharmacoeconomic Evaluation and varies among countries. This present study analyzed the cost-effectiveness of PFO closure vs. medical therapy alone to guide Chinese doctors in their choice of treatment plans for CS patients with PFO.

This study aimed to evaluate the cost-effectiveness of percutaneous device-based PFO closure vs. medical therapy alone in CS patients with PFO from the perspective of medical payers. Owing to disability and a substantial reduction in income, young and middle-aged patients with stroke incur higher medical costs for the rest of their lives (15, 16). Hence, an appropriate therapeutic method to decrease stroke recurrence is of great significance in these populations.

## Methods

### Model overview

**Figure 1:**
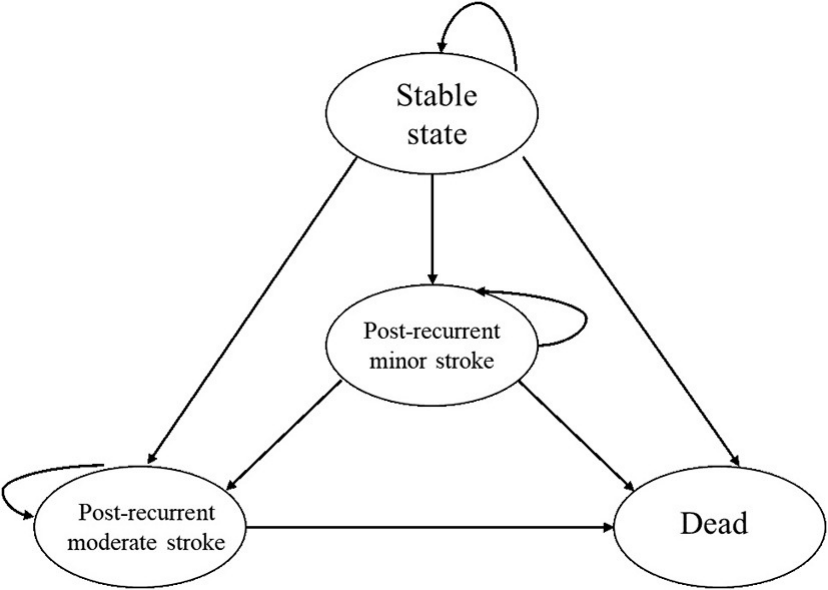
Possible state transition in the Markov model with a cycle length of 3 months.

We used a Markov model to compare the long-term cost and effectiveness of percutaneous PFO closure vs. medical therapy alone based on a previously published model submitted to the UK National Institute for Health and Care Excellence in 2017 (17). In brief, there were four health states in the model, including “Stable state,” “Post-recurrent minor stroke,” “Post-recurrent moderate stroke,” and “Dead.” A minor stroke was defined as the Modified Rankin Scale (mRS) score of ≤2, and a moderate stroke was defined as an mRS score of ≥3. The cycle length of the model was 3 months, and patients would be cycled in the model until death or the time horizon of the model, which was set at 30 years. The details of the model are shown in Figure 1.

All analyses were performed using TreeAge Pro 2011 software (TreeAge Software, Inc., Williamstown, MA, USA). Additionally, a half-cycle correction was used to avoid overestimating cost and effectiveness (18).

### Population

The target population of the present study was a hypothesis cohort in China with similar baseline characteristics to those in the RESPECT study (15). In the RESPECT study, the patients had a mean age of 46 years, ranging from 18 to 60 years, had a cryptogenic ischemic stroke, and PFO was confirmed by transesophageal echocardiography. They were randomly allocated to receive percutaneous PFO closure or medical therapy alone and were followed up for 5.9 years. In the PFO closure cohort, an Amplatzer PFO Occluder was implanted in patients with PFO, and 81–325 mg of aspirin plus 75 mg of clopidogrel was prescribed to be taken daily for one month after the procedure, followed by 100 mg of aspirin monotherapy for 5 months. The patients received aspirin, warfarin, clopidogrel, or aspirin combined with extended-release dipyridamole in the medical therapy cohort.

### Input parameters

All the input parameters used for base case analysis are shown in Table 1.

**Table 1:** Input parameters of transition probability, utility, and cost in the base case analysis.

**Parameters**	**Base value**	**Lower value**	**Higher value**	**Distribution**	**References**
**Transition probability (per 3 months)**					
Recurrent ischemic stroke in PFO closure^a^	0.00145	0.00079	0.00212	β	(15)
Recurrent ischemic stroke in Medical Therapy^a^	0.00269	0.0017	0.00367	β	(15)
**The proportion of patients with recurrent stroke**					
Minor stroke (mRS ≤ 2)	0.534	0.470	0.596	/	(19)
Moderate stroke (mRS 3–5)	0.324	0.267	0.386	/	(19)
Death (mRS 6)	0.142	0.102	0.192	/	(19)
**Three-month mortality of the general population**					
45–50	0.00048	/	/	/	(20)
50–55	0.00076	/	/	/	(20)
55–60	0.00121	/	/	/	(20)
60–65	0.00193	/	/	/	(20)
65–70	0.00325	/	/	/	(20)
70–75	0.00565	/	/	/	(20)
75–80	0.00991	/	/	/	(20)
The relative risk of non-stroke death for moderate stroke	1.68	1.49	1.91	/	(19)
**Utility for post-stroke**					
Minor stroke	0.76	0.69	0.82	β	(19, 21)
Moderate stroke	0.21	0.17	0.26	β	(19, 21)
Recurrent stroke	0.2	0.16	0.26	β	(19, 21)
**Utility for a stable state**					
Medical therapy	0.80	0.72	0.88	β	(17)
Within 6 months post-PFO closure	0.84	0.80	0.88	β	(17)
After 6 months post-PFO closure	0.88	0.79	0.97	β	(17)
**Cost (USD)**					
PFO closure device^b^	4938	1451	5926	γ	Institutional data
PFO closure procedure	517	459	574	γ	Institutional data
Other costs of PFO closure	1180	611	1749	γ	Institutional data
Minor stroke event^c^	1901	950	3802	γ	(22)
Moderate stroke event^d^	2513	1257	5026	γ	(22)
Stroke death^e^	2154	1077	4308	γ	(22)
Quarterly cost of post-minor stroke^f^	338	169	676	γ	(22)
Quarterly cost of post-moderate stroke^g^	514	257	1028	γ	(22)
Aspirin (100 mg, 30 tablets)	2.3	1.2	4.7	γ	(23)
Clopidogrel (75 mg, 28 tablets)	14.6	7.3	29.1	γ	(23)
Discount rate	0.05	0	0.08	/	(24)

PFO, patent foramen ovale; CNY, Chinese Yuan.

a Recurrent ischemic stroke rate per 100 patient-year is 0.58% in PFO closure cohort and 1.07 in medical therapy cohort, the 3-month rate is 0.00145422133039121 = –ln (1–0.58%)/4 in PFO closure cohort and 0.00268941416324005 = –ln (1–1.07%)/4, then the transition probability in two cohorts is 0.00145316446292076 = 1 – exp(−0.00145422133039121) and 0.0026858009288564 = 1 – exp(−0.00268941416324005), separately.

b The cost of the Amplatzer PFO Occluder is CNY ¥31,860 (US $4,938) in China.

c The cost of a minor stroke event is CNY ¥12,214 (US $1,893) in 2020, and it is US $1,901 in 2021, calculated as follows: 12214^*^1.004/6.4515.

d The calculation method is the same as that in note 3.

e The calculation method is the same as that in note 3.

f The annual cost of post-minor stroke is CNY ¥8,684 (US $1,346) in 2020, and the quarterly cost is US $338, which is calculated as follows: 8684^*^1.004/4/6.4515.

g The calculation method is the same as in note 6.

#### Transition probability

The transition probability of recurrent stroke was derived from the RESPECT study, as there are no reports on the incidence of recurrent stroke in Chinese patients with CS and PFO. In the RESPECT study, the rate of recurrent stroke was 0.58 per 100 patient-year in the PFO closure cohort and 1.07 in the medical therapy cohort. The transition probability was assumed to be constant for every cycle. Thus, we can conclude that the 3-month rate of recurrent stroke using the formula of “3-month rate = -ln (1 – 1-year rate)/4,” and the 3-month transition probability is “3-month transition probability = 1 – exp (-3-month rate)” (25). The transition probability included both minor and moderate stroke cases, and the proportion of minor stroke was ~53.4% of all strokes, while the proportion of moderate stroke was 32.4% and that of death was 14.2%. The proportion of patients with recurrent stroke was derived from an RCT investigating the quality improvement intervention for acute ischemic stroke in China (19, 26). Background mortality was accessed from the China Health Statistical Yearbook 2021 (20) and was age-dependent, with a gap of 5 years. For patients with moderate stroke, a risk ratio of 1.68 (95% CI 1.49, 1.91) was employed to adjust for non-stroke death (27, 28).

#### Cost

The cost of PFO closure was obtained from three hospitals located at different sites to represent the overall cost of PFO closure in China. The cost of PFO closure includes three parts: the device, the procedure, and other costs. In our analysis, all the costs not denominated in Chinese Yuan (CNY) were converted to CNY in 2021, taking the consumer price index (CPI) into consideration; the CPIs for the healthcare sector in China from 2015 to 2021 were 1.027, 1.038, 1.06, 1.043, 1.024, 1.018, and 1.004, respectively. Subsequently, CNY was converted to USD, with an average exchange ratio of 6.4515. The cost of the PFO closure device in the base case analysis was US $4,938, which was the cost of the Amplatzer PFO Occluder, as the transition probability in our analysis was derived from the RESPECT study where the Amplatzer PFO Occluder was used. The mean cost of the PFO closure procedure was US $517, and the other cost was US $1,180. The stroke-related cost was obtained from a published study that performed an economic evaluation of intravenous alteplase for stroke in the Chinese population (22). In this study, the costs of minor and moderate stroke events were CNY ¥12214 (US $1,893) and CNY ¥16149 (US $2503), respectively. When taking into consideration the healthcare CPI, the cost would be US $1,901 and US $2,513, and we could obtain the quarterly cost of post-minor stroke and moderate stroke as US $338 and US $514, respectively.

Regarding the cost of antithrombotic medications, the cost of 100 mg of aspirin (30 tablets) was CNY ¥15 (US $2.3), while the cost of 75 mg of clopidogrel (28 tablets) was CNY ¥94 (US $14.6). The cost of 3 mg of warfarin, 20 mg of rivaroxaban, and 110 mg of dabigatran was CNY ¥0.5 CNY (US $0.08), CNY ¥27 (US $4.2), and CNY ¥14 (US $2.2), respectively (23). The cost of the above antithrombotic medications was almost uniform across the country and was available to patients *via* various methods, including at hospitals, at pharmacies, and online.

#### Utility

The utility was obtained from a domestic study investigating stroke's utility in Chinese patients. They found that the utility was 0.76 (95% CI 0.69, 0.82), 0.21 (95% CI 0.17, 0.26), and 0.20 (95% CI 0.16, 0.26) for minor, moderate, and recurrent strokes, respectively. For utilities post-procedure, the utility is 0.84 (0.80, 0.88) for the stable state within 6 months, 0.88 (0.79, 0.97) for the stable state after 6 months, and 0.80 (0.72, 0.88) for those who received medical therapy (17).

All input parameters, except transition probability, including cost and utility, were discounted at a rate of 0.05, in the range of 0.0–0.08, according to the China Guidelines for Pharmacoeconomic Evaluations (24).

### Outcome

The primary outcome of this study was the ICER, which represents the incremental cost per incremental quality-adjusted life year (QALY). Since the Chinese government recommends no specific WTP threshold, we adopted three times the per capita GDP of China in 2021 as the WTP threshold, as recommended by the China Guidelines for Pharmacoeconomic Evaluations (24). The per capita GDP in China in 2021 was 12551.3 USD, so the WTP was set at 37,654 USD/QALY. PFO closure was considered cost-effective if the ICER obtained was lower than the WTP threshold; otherwise, PFO closure was regarded as not being cost-effective. The secondary outcomes included overall cost, overall effectiveness, incremental cost, incremental effectiveness, ICER of incremental cost per incremental life year, and ICER based on different time horizons.

### Scenario and sensitivity analysis

A scenario analysis was performed based on different antithrombotic medications in medical therapy and the utility measured in another study (Table 2).

**Table 2:** Input parameters in the scenario analysis.

**Scenario 1: Other medications in medical therapy**	**Value**	**References**
Cost of warfarin (USD/tablet)	0.08	(23)
Annual frequency of INR examination^a^	21	(23)
Cost of INR examination^a^	12	(23)
Cost of dabigatran (CNY/110 mg, tablet)	2.2	(23)
Cost of rivaroxaban (CNY/20 mg, tablet)	4.2	(23)
**Scenario 2: Utility measured with SF-6D published in another study**		
Minor stroke	0.8	(29)
Moderate stroke	0.59	(29)
Utility for the stable state in medical therapy	0.87	(30)
Utility for a stable state within 6 months post-PFO closure	0.91	(30)
Utility for stable state after 6 months post-PFO closure	0.96	(30)
**Scenario 3: Utility measured with EQ-5D published in another study**		
Post-minor stroke	0.87	(29)
Post-moderate stroke	0.51	(29)
Utility for the stable state in medical therapy	0.87	(30)
Utility for a stable state within 6 months post-PFO closure	0.91	(30)
Utility for stable state after 6 months post-PFO closure	0.96	(30)

a Those who take warfarin need to have an examination of the international normalized ratio (INR); the cost of the INR examination is 75 CNY (12 USD)/time, with a frequency of 21 times/year.

One-way sensitivity analysis and probabilistic sensitivity analysis (PSA) were performed to test the robustness of the results. In the one-way sensitivity analysis, the input parameters fluctuated within their 95% confidence intervals or given intervals. For the cost of the PFO closure device, the lower range adopted the lowest cost of the PFO closure device, and the higher range adopted 1.2 times the cost of the Amplatzer PFO Occluder. We adopted a broader range for the stroke-related cost because the PFO-caused stroke might differ slightly from the other-cause-related stroke. A tornado diagram of the ICER was used to illustrate the results of the one-way sensitivity analysis. In PSA, the utility and transition probability parameters followed the β distribution, and the cost parameter followed the γ distribution. Probabilistic sensitivity samplings based on 10,000 times Monte Carlo simulations were employed, and the results of the PSA were illustrated with a scatter plot and an acceptability curve.

## Results

### Outcomes

As shown in Table 3, after a simulation of 30 years, a cryptogenic stroke patient with PFO is expected to have QALY of 13.15 (15.26 LY) if he receives PFO closure and 11.74 QALY (15.14 LY) after medical therapy alone. The corresponding costs in both cohorts are US $8,131 and US $4,186, respectively. An ICER of US $2,783/QALY and US $31,264/LY was obtained, which is lower than the WTP threshold.

**Table 3:** Base case analysis and scenario analysis.

**Strategy**	**Cost (USD)**	**Incre-Cost (USD)**	**Eff (QALY)**	**Incre-Eff (QALY)**	**ICER (USD/QALY)**	**Eff (LY)**	**Incre-Eff (LY)**	**ICER (USD/LY)**
**Base case analysis**								
Medical therapy	4186	–	11.74	–	–	15.14	–	–
PFO closure	8131	3945	13.15	1.42	2783	15.26	0.13	31264
**Scenario analysis**								
Warfarin in medical therapy								
Medical therapy	6352	–	11.74	–	–	15.14	–	–
PFO closure	8131	1779	13.15	1.42	1255	15.26	0.13	14098
Dabigatran in medical therapy								
Medical therapy	24085	–	11.74	–	–	15.14	–	–
PFO closure	8131	−15954	13.15	1.42	−11256	15.26	0.13	−126433
Rivaroxaban in medical therapy								
Medical therapy	23318	–	11.74	–	–	15.14	–	–
PFO closure	8131	−15187	13.15	1.42	−10715	15.26	0.13	−120356
Utility measured with SF−6D published in another study								
Medical therapy	4186	–	12.93	–	–	15.14	–	–
PFO closure	8131	3945	14.44	1.51	2617	15.26	0.13	31264
Utility measured with EQ−5D published in another study								
Medical therapy	4186	–	12.95	–	–	15.14	–	–
PFO closure	8131	3945	14.45	1.50	2628	15.26	0.13	31264

Incre, incremental; Eff, effectiveness; QALY, quality-adjusted life year; ICER, incremental cost-effectiveness ratio; LY, life year; and PFO, patent foramen ovale.

**Figure 2:**
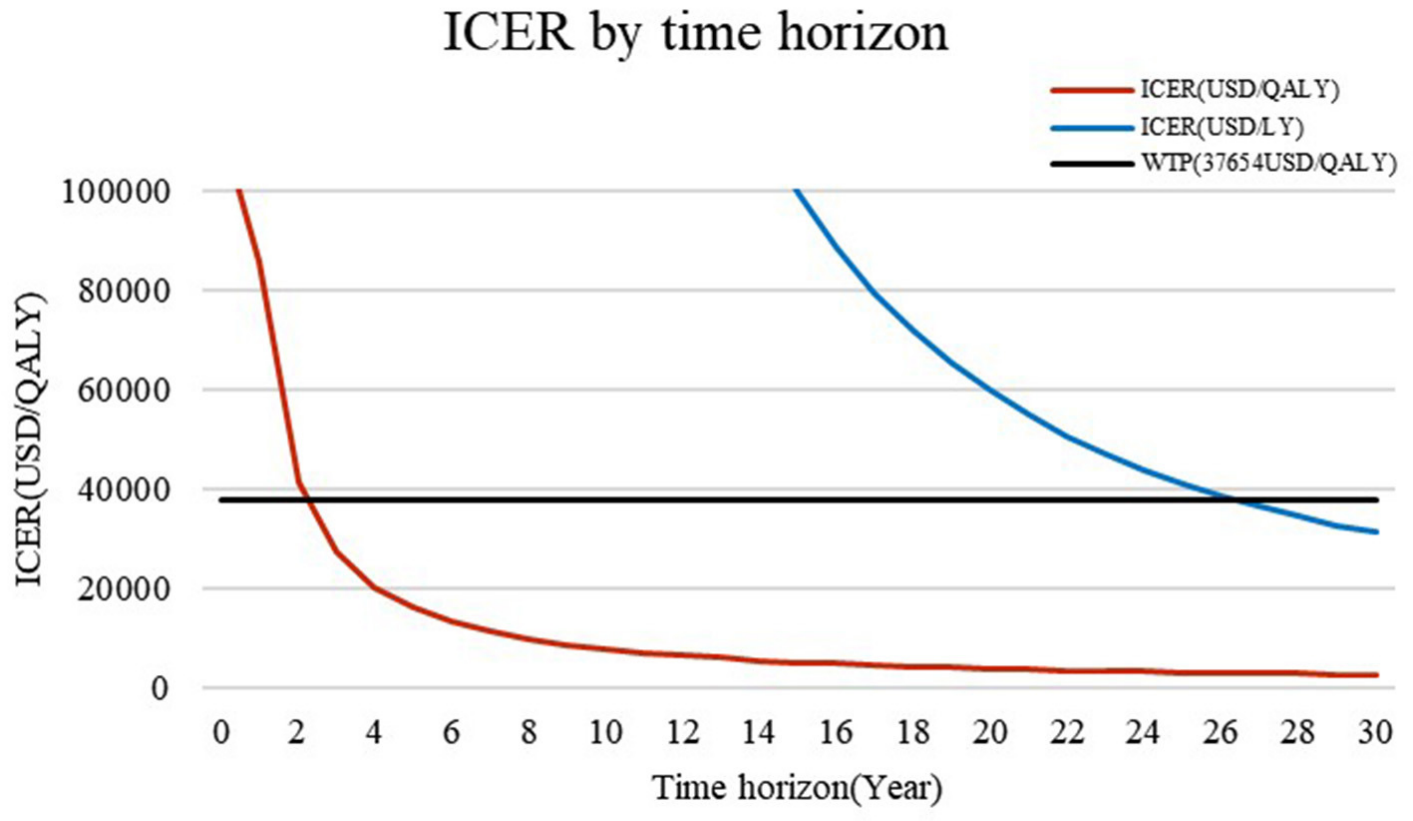
ICER based on different time horizons. When the time horizon is <3 years, the ICER is higher than the WTP of US $37,654/QALY. However, if the quality of life is not considered, PFO is cost-effective for those with a life expectancy of >26 years.

The ICER based on different time horizons shows that, when life expectancy is over 2 years, PFO closure is cost-effective when the quality of life is taken into consideration, whereas life expectancy needs to be at least 26 years for PFO closure to be cost-effective when quality is not taken into consideration (Figure 2).

### Scenario analysis

Scenario analysis based on different antithrombotic medications in medical therapy and utility measured in another study showed that PFO closure is cost-effective or superior to medical therapy in Chinese cryptogenic stroke patients with PFO (Table 3).

### Sensitivity analysis

**Figure 3:**
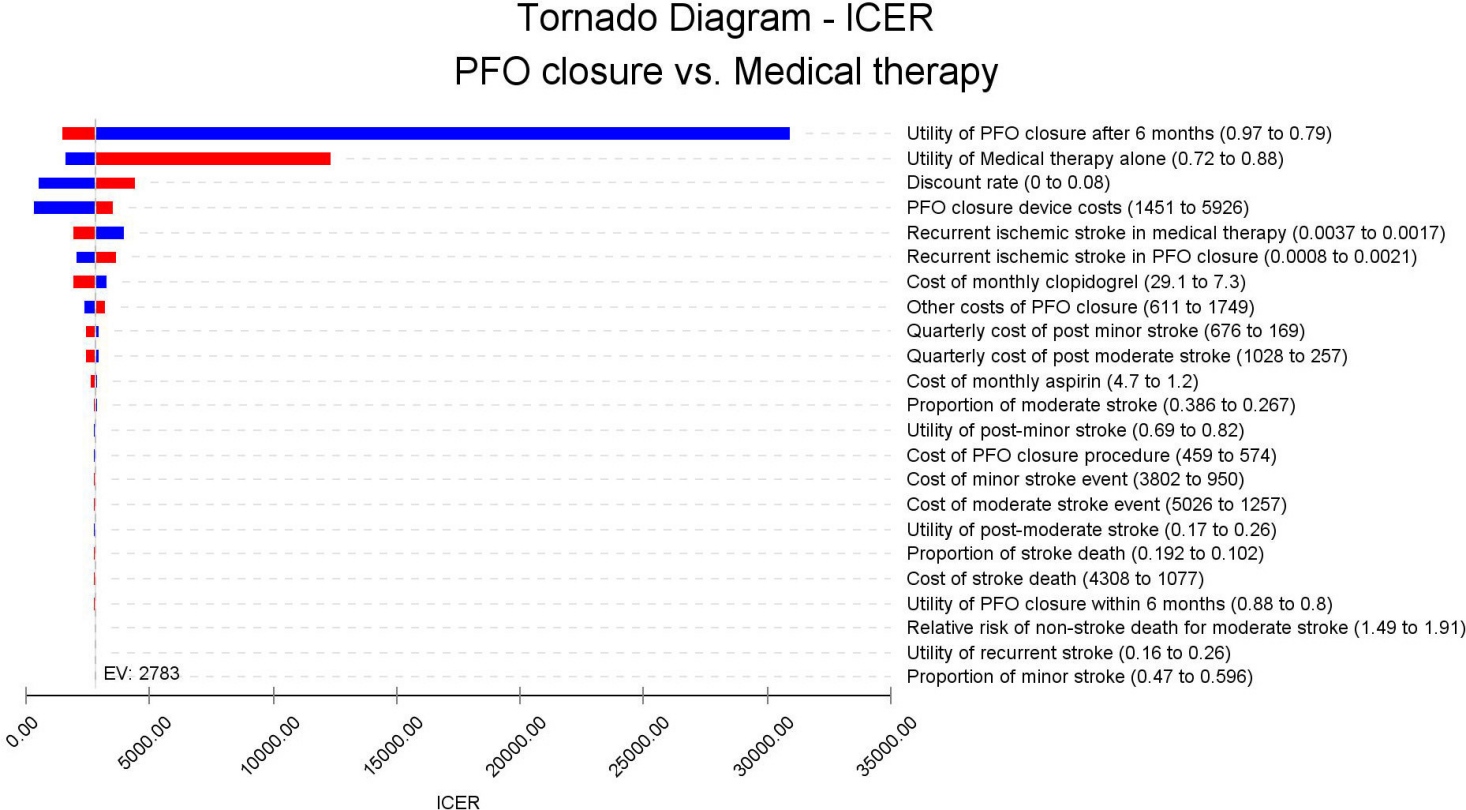
Tornado diagram: ICER of PFO closure vs. medical therapy according to different model inputs. The ICER of base case analysis lies in the middle of the tornado diagram; the left and right sides of each bar in the tornado graph represent the ICER value with the lower parameter input or higher parameters input (according to the parameter description of the tornado diagram).

One-way sensitivity analysis showed that the utility of PFO closure after 6 months had the largest impact on the ICER fluctuation. However, China's highest ICER was still lower than the WTP threshold of 37,654 USD/QALY (Figure 3).

**Figure 4:**
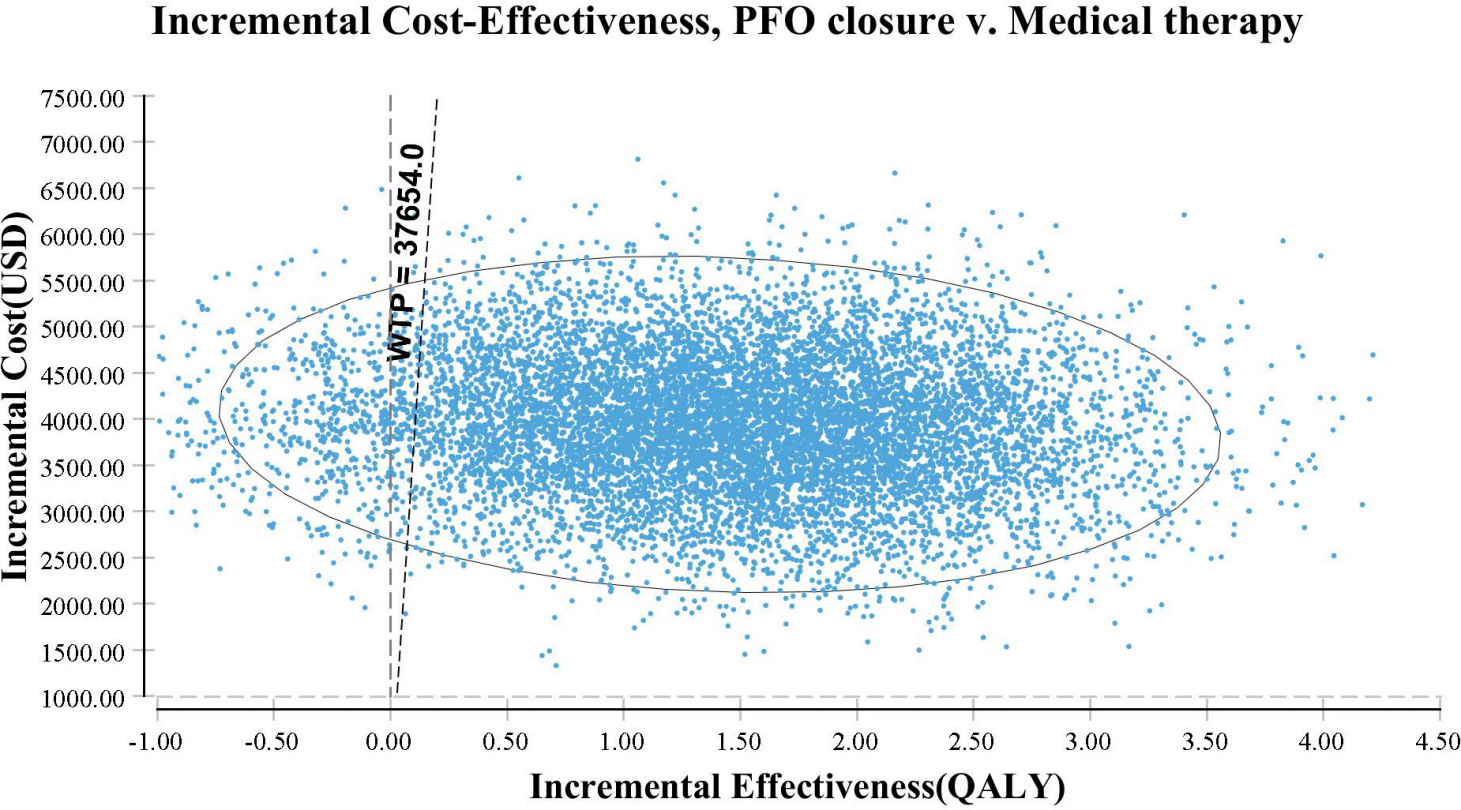
A scatter plot based on probabilistic sensitivity analyses. The straight line represents the willingness-to-pay threshold of US $37,654/QALY.

**Figure 5:**
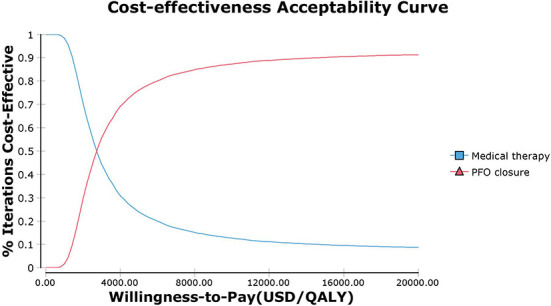
Cost-effectiveness acceptability curve. PFO closure and medical therapy share similar acceptability when the WTP threshold is US $2,700/QALY. At the current WTP threshold of US $37,654/QALY in China, PFO closure is more acceptable than medical therapy alone.

The PSA results show that PFO closure is cost-effective when the probability is over 92% (Figure 4). The acceptability curve shows that, when the WTP threshold is ~US $2,700 /QALY, which is far lower than the WTP threshold in China, PFO closure shares similar acceptability with medical therapy alone (Figure 5).

## Discussion

PFO closure has been proven as an effective way to reduce recurrent stroke in cryptogenic stroke patients with PFO and with a hazard ratio of 0.55 (95% CI 0.31–0.999). Although the absolute risk reduction is 0.49% per patient-year, which means that, for 100 patients with a follow-up period of 1 year, there will be 0.49 fewer events in the PFO closure than in medical therapy alone (15), it is necessary to investigate the cost-effectiveness of PFO closure vs. medical therapy alone.

Shijoh et al. found that the probability of cost-effective PFO closure with Monte Carlo simulation was 50.3% at a WTP threshold of US $50,000/QALY (31). This WTP threshold is higher than the estimated WTP threshold in China, and it can be assumed that PFO closure will not be cost-effective if the ICER in Shijoh's study is employed in China. In contrast, we found that PFO closure is cost-effective with over 92% probability in our study, and this may be due to the following reasons: First, the PFO closure device and procedural cost is US $16,109 in Japan (31) but US $4,738 in China (32), which is <30% of the cost in Japan. Second, the simulation period was longer in our study than in Shijoh's study, and we know that the benefit of PFO closure could last for a long time (33, 34) and that a longer simulation period will result in a lower ICER and higher acceptability. Notably, PFO closure is cost-effective when the life expectancy is 2 years in our study, which is driven mainly by the fact that the overall cost of PFO closure is low. Patients could be more effective if their quality of life improved. Another study investigating the lifetime cost-effectiveness of PFO closure vs. medical therapy alone found that PFO closure is dominant compared to medical therapy in Japan (30). In their study, the effectiveness gained in PFO closure was higher than ours, which might be partly due to the higher life expectancy in Japan. Moreover, their finding that PFO closure costs less than medical therapy can be attributed to the high cost of medical therapy. In our scenario analysis, we found that, if the patients in the medical therapy cohort received direct oral anticoagulation agents (the cost of direct oral anticoagulation agents is higher than that of aspirin, clopidogrel, and warfarin) (23), PFO closure would be dominant for the same reason.

Our study also shows that ICER will reduce with time. Some studies conducted in other settings have drawn a similar conclusion that a longer time horizon results in a lower ICER. Volpi et al. found that, when the time horizon is extended from 3 years to 20 years, the ICER decreases from US $35,755/QALY to US $1,894/QALY (35), and this finding has been validated by several studies (17, 36–38). These results, therefore, suggest that PFO may be suitable for patients with long life expectancies.

In the scenario analysis, the utilities adopted from another study have little impact on the results. However, different antithrombotic medications in medical therapy may cause medical therapy to be inferior to PFO closure, and this is due to the high cost of direct oral anticoagulation agents. The annual cost of direct oral anticoagulation agents is US $1,507 (23, 32), which is almost one-fourth of the overall cost of PFO closure.

Other factors also had little impact on the fluctuation of the ICER. In the one-way sensitivity analysis, the utility of PFO closure after 6 months and the utility of medical therapy alone had the largest impact on the ICER; however, the ICER obtained was still lower than the WTP threshold. Even when the quality of life is not considered, PFO closure is still cost-effective. The scatter plot and the acceptability curve confirm the robustness of the results.

Our study has some limitations. First, the present study is based on a mathematical model. Even though one-way and probabilistic sensitivity analyses confirmed the robustness of the findings, a real-world study may provide more clinical evidence. Second, the study is conducted from the perspective of healthcare providers, and this perspective may not provide the most comprehensive view; a perspective from society may be better. Third, the transition probability in our analysis was derived from the RESPECT study, which is the largest RCT with the longest follow-up period. As there is no study investigating the effectiveness of PFO closure vs. medical therapy in Chinese patients, the data from Chinese patients might have provided more accurate information. Fourth, procedure-related complications, including new-onset atrial fibrillation and residual shunts, were not included in the analysis, which is another significant weakness of our model. Fifth, the costs and utilities in the model were derived from stroke patients rather than those with PFO, which might have introduced bias to some extent. Lastly, the follow-up period in the RESPECT study was 5.9 years. In contrast, the time horizon in our study was far longer than that, which might have introduced more uncertainty, as PFO closure has been shown to be cost-effective in the third year, and the 30-year horizon may not influence the robustness of our results.

In conclusion, with respect to the WTP threshold of three times the per capita GDP in China in 2021, PFO closure is a cost-effective method for treating Chinese cryptogenic stroke patients with PFO, as we have shown in the 30-year simulation.

## Data availability statement

The original contributions presented in the study are included in the article/supplementary material, further inquiries can be directed to the corresponding author/s.

## Author contributions

FL and XY proposed and designed this study. NW developed the model and wrote the manuscript. BL, MM, XZ, WZ, and FH identified the cost and utility parameters. All authors contributed to the article and approved the submitted version.

## Funding

This study was supported by Natural Science Foundation of Shaanxi Province (Nos. 2022SF-476 and 2021SF-329), the Science and Technology Program of Xi'an (No. 21YXYJ0095), Key Industrial Innovation Chain Project in Shaanxi Province of China (No. 2021ZDLSF02-03), Shaanxi Provincial Health and Health Research Fund Project (No. 2022D024), and the Key Basic Natural Science Foundation of Shaanxi Province (No. 2022JZ-47).

## Conflict of interest

The authors declare that the research was conducted in the absence of any commercial or financial relationships that could be construed as a potential conflict of interest.

## Publisher's note

All claims expressed in this article are solely those of the authors and do not necessarily represent those of their affiliated organizations, or those of the publisher, the editors and the reviewers. Any product that may be evaluated in this article, or claim that may be made by its manufacturer, is not guaranteed or endorsed by the publisher.

## References

[1] Virani SS Alonso A Aparicio HJ Benjamin EJ Bittencourt MS Callaway CW. Heart disease and stroke statistics-2021 update: a report from the American heart association. Circulation. (2021) 143:e254–743. 10.1161/CIR.0000000000000950 33501848 PMC13036842

[2] Krishnamurthi RV Feigin VL Forouzanfar MH Mensah GA Connor M Bennett DA. Global and regional burden of first-ever ischaemic and h stroke during 1990–2010: findings from the global burden of disease study 2010. Lancet Glob Health. (2013) 1:e259–81. 10.1016/S2214-109X(13)70089-5 25104492 PMC4181351

[3] Benjamin EJ Blaha MJ Chiuve SE Cushman M Das SR Deo R. Heart disease and stroke statistics-2017 update: a report from the American heart association. Circulation. (2017) 135:e146–603. 10.1161/CIR.0000000000000491 28122885 PMC5408160

[4] Wang YJ Li ZX Gu HQ Zhai Y Zhou Q Jiang Y. China stroke statistics: an update on the 2019 report from the national center for healthcare quality management in neurological diseases, china national clinical research center for neurological diseases, the Chinese stroke association, national center for chronic and non-communicable disease control and prevention, Chinese center for disease control and prevention and institute for global neuroscience and stroke collaborations. Stroke Vascular Neurol. (2022) svn-2021. 10.1136/svn-2021-001374 [Epub ahead of print]. 35443985 PMC9614174

[5] Sanna T Diener HC Passman RS Di Lazzaro V Bernstein RA Morillo CA. Cryptogenic stroke and underlying atrial fibrillation. N Engl J Med. (2014) 370:2478–86. 10.1056/NEJMoa1313600 24963567

[6] Zhang YS Zhu XY Kong XQ Jiang SL. Chinese expert consensus on preventive closure of patent foramen ovale. Chinese Circulat J. (2017) 32:209–14. 10.3969/j.issn.1000-3614.2017.03.001

[7] Liberman AL Prabhakaran S. Cryptogenic stroke: how to define it? How to treat it? Curr Cardiol Rep. (2013) 15:423. 10.1007/s11886-013-0423-x 24105642

[8] Saver JL. Clinical practice. Cryptogenic stroke. N Engl J Med. (2016) 374:2065–74. 10.1056/NEJMcp1503946 27223148

[9] Alsheikh-Ali AA Thaler DE Kent DM. Patent foramen ovale in cryptogenic stroke: incidental or pathogenic? Stroke. (2009) 40:2349–55. 10.1161/STROKEAHA.109.547828 19443800 PMC2764355

[10] Saver JL Mattle HP Thaler D. Patent foramen ovale closure vs. medical therapy for cryptogenic ischemic stroke: a topical review. Stroke. (2018) 49:1541–8. 10.1161/STROKEAHA.117.018153 29760277

[11] Tsivgoulis G Katsanos AH Mavridis D Frogoudaki A Vrettou AR Ikonomidis I. Percutaneous patent foramen ovale closure for secondary stroke prevention: network meta-analysis. Neurology. (2018) 91:e8–e18. 10.1212/WNL.0000000000005739 29875217

[12] Zhang C Zheng L Zhang Y Guo H Chi J. Comparison of stroke recurrence between antiplatelet and closure therapy in cryptogenic stroke patients with patent foramen ovale and its influencing factors. Eur J Intern Med. (2020) 78:149–51. 10.1016/j.ejim.2020.04.007 32307231

[13] He L Cheng GS Du YJ Zhang YS. Multidisciplinary assessment of PFO with substantial right-to-left shunting and medium-term follow-up after PFO device closure: a single-center experience. J Interv Cardiol. (2017) 30:362–7. 10.1111/joic.12396 28568903 PMC5575516

[14] Ma LY Wang ZW Fan J Hu SS. Report on cardiovascular health and diseases in China 2021: an updated summary. Chinese Circulat. J. (2022) 37:553–78. 10.3969/j.issn.1000-3614.2022.06.00135945174

[15] Saver JL Carroll JD Thaler DE Smalling RW MacDonald LA Marks DS. Long-term outcomes of patent foramen ovale closure or medical therapy after stroke. N Engl J Med. (2017) 377:1022–32. 10.1056/NEJMoa1610057 28902590

[16] Kitsios GD Dahabreh IJ Abu Dabrh AM Thaler DE Kent DM. Patent foramen ovale closure and medical treatments for secondary stroke prevention: a systematic review of observational and randomized evidence. Stroke. (2012) 43:422–31. 10.1161/STROKEAHA.111.631648 22180252 PMC3342835

[17] Tirschwell DL Turner M Thaler D Choulerton J Marks D Carroll J. Cost-effectiveness of percutaneous patent foramen ovale closure as secondary stroke prevention. J Med Econ. (2018) 21:656–65. 10.1080/13696998.2018.1456445 29564942

[18] Sonnenberg FA Beck JR. Markov models in medical decision making: a practical guide. Med Decis Making. (1993) 13:322–38. 10.1177/0272989X9301300409 8246705

[19] Pan Y Zhang L Li Z Meng X Wang Y Li H. Cost-effectiveness of a multifaceted quality improvement intervention for acute ischemic stroke in China. Stroke. (2020) 51:1265–71. 10.1161/STROKEAHA.119.027980 32019480

[20] Wu SY Yu SL Lei ZL Meng Q Li TR Xu ZY. China Health Statistics Yearbook 2021. 2021 ed. Beijing: Peking Union Medical College Press (2021).

[21] Wang YL Pan YS Zhao XQ Wang D Johnston SC Liu LP. Recurrent stroke was associated with poor quality of life in patients with transient ischemic attack or minor stroke: finding from the CHANCE trial. CNS Neurosci Ther. (2014) 20:1029–35. 10.1111/cns.12329 25307297 PMC6493002

[22] Chen J Liang X Tong X Han M Ji L Zhao S. Economic evaluation of intravenous alteplase for stroke with the time of onset between 4.5 and 9 hours. J Neurointerv Surg. (2022) 1–7. 10.1136/neurintsurg-2021-018420 [Epub ahead of print]. 35074896 PMC9763196

[23] Wei H Cui C Cui X Liu Y Li D. Cost-effectiveness analysis of dabigatran, rivaroxaban and warfarin in the prevention of stroke in patients with atrial fibrillation in China. BMC Health Serv Res. (2021) 21:96. 10.1186/s12913-021-06084-1 33509171 PMC7841891

[24] Liu GG Hu SL Wu JH Wu J Yang L Li HC. China Guidelines for Pharmacoeconomic Evaluations, Chinese-English Version. Beijing: China Market Press (2020).

[25] Krittayaphong R Permsuwan U. Cost-utility analysis of sacubitril-valsartan compared with enalapril treatment in patients with acute decompensated heart failure in Thailand. Clin Drug Invest. (2021) 41:907–15. 10.1007/s40261-021-01079-6 34533783 PMC8446182

[26] Wang Y Li Z Zhao X Wang C Wang X Wang D. Effect of a multifaceted quality improvement intervention on hospital personnel adherence to performance measures in patients with acute ischemic stroke in china: a randomized clinical trial. JAMA. (2018) 320:245–54. 10.1001/jama.2018.8802 29959443

[27] Slot KB Berge E Sandercock P Lewis SC Dorman P Dennis M. Causes of death by level of dependency at 6 months after ischemic stroke in 3 large cohorts. Stroke. (2009) 40:1585–9. 10.1161/STROKEAHA.108.531533 19265057

[28] Samsa GP Reutter RA Parmigiani G Ancukiewicz M Abrahamse P Lipscomb J. Performing cost-effectiveness analysis by integrating randomized trial data with a comprehensive decision model: application to treatment of acute ischemic stroke. J Clin Epidemiol. (1999) 52:259–71. 10.1016/S0895-4356(98)00151-6 10210244

[29] Du XD Zhu P Li ME Wang J Meng HD Zhu CR. Health utility of patients with stroke measured by EQ-5D and SF-6D. J Sichuan Univ Med Sci Edn. (2018) 49:252–7. 10.13464/j.scuxbyxb.2018.02.020 29737071

[30] Inoue S Igarashi A Iguchi Y Akagi T. Cost-effectiveness analysis of percutaneous patent foramen ovale closure preventing secondary ischemic stroke in Japan. J Stroke Cerebrovascular Dis Official J Natl Stroke Assoc. (2021) 30:105884. 10.1016/j.jstrokecerebrovasdis.2021.105884 34153592

[31] Shijoh Y Saito S Dai Z Ohde S. Cost-effectiveness analysis of patent foramen ovale closure vs. medical therapy alone after cryptogenic stroke. PLoS ONE. (2022) 17:e0268690. 10.1371/journal.pone.0268690 35657973 PMC9165785

[32] Han Y. Clinical Study on Patent Foramen Ovale Closure Under Only Echocardiography Guidance for Cryptogenic Stroke, in Department of Surgery. Jinan: Shandong University (2020). p. 134.

[33] Abdelaziz HK Saad M Abuomara HZ Nairooz R Pothineni NV Madmani ME et al. Long-term outcomes of patent foramen ovale closure or medical therapy after cryptogenic stroke: a meta-analysis of randomized trials. Catheter Cardiovasc Interv. (2018) 92:176–86. 10.1002/ccd.27636 29726616

[34] Ateş AH Yorgun H Canpolat U Sener YZ Okşul M Kaya EB. Long-term follow-up outcomes in a real-world study cohort after percutaneous patent foramen ovale closure. Turk Kardiyol Dern Ars. (2021) 49:29–39. 10.5543/tkda.2020.06699 33390571

[35] Volpi JJ Ridge JR Nakum M Rhodes JF Søndergaard L Kasner SE. Cost-effectiveness of percutaneous closure of a patent foramen ovale compared with medical management in patients with a cryptogenic stroke: from the US payer perspective. J Med Econ. (2019) 22:883–90. 10.1080/13696998.2019.1611587 31025589

[36] Hildick-Smith D Turner M Shaw L Nakum M Hartaigh BÓ Evans RM. Evaluating the cost-effectiveness of percutaneous closure of a patent foramen ovale vs. medical management in patients with a cryptogenic stroke: from the UK payer perspective. J Med Econ. (2019) 22:131–9. 10.1080/13696998.2018.1548355 30424680

[37] Pickett CA Villines TC Resar JR Hulten EA. Cost effectiveness and clinical efficacy of patent foramen ovale closure as compared to medical therapy in cryptogenic stroke patients: A detailed cost analysis and meta-analysis of randomized controlled trials. Int J Cardiol. (2018) 273:74–9. 10.1016/j.ijcard.2018.07.099 30119914

[38] Pickett CA Villines TC Ferguson MA Hulten EA. Cost effectiveness of percutaneous closure vs. medical therapy for cryptogenic stroke in patients with a patent foramen ovale. Am J Cardiol. (2014) 114:1584–9. 10.1016/j.amjcard.2014.08.027 25248812

